# ASXL1 plays an important role in erythropoiesis

**DOI:** 10.1038/srep28789

**Published:** 2016-06-29

**Authors:** Hui Shi, Shohei Yamamoto, Mengyao Sheng, Jie Bai, Peng Zhang, Runze Chen, Shi Chen, Lihong Shi, Omar Abdel-Wahab, Mingjiang Xu, Yuan Zhou, Feng-Chun Yang

**Affiliations:** 1 Sylvester Comprehensive Cancer Center, University of Miami Miller School of Medicine, Miami, FL 33136, USA; 2 Department of Biochemistry and Molecular Biology, University of Miami Miller School of Medicine, Miami, FL 33136, USA; 3 State Key Laboratory of Experimental Hematology, Institute of Hematology & Blood Diseases Hospital, Center for Stem Cell Medicine, Chinese Academy of Medical Sciences & Peking Union Medical College, Tianjin 300020, China; 4 Showa University Fujigaoka Hospital, 1-30 Fujigaoka Aoba-Ku, Yokohamashi Kanagawa, 227-8501, Japan; 5 Human Oncology and Pathogenesis Program, Memorial Sloan Kettering Cancer Center, New York, NY 10065, USA

## Abstract

*ASXL1* mutations are found in a spectrum of myeloid malignancies with poor prognosis. Recently, we reported that *Asxl1*^+/*−*^ mice develop myelodysplastic syndrome (MDS) or MDS and myeloproliferative neoplasms (MPN) overlapping diseases (MDS/MPN). Although defective erythroid maturation and anemia are associated with the prognosis of patients with MDS or MDS/MPN, the role of ASXL1 in erythropoiesis remains unclear. Here, we showed that chronic myelomonocytic leukemia (CMML) patients with *ASXL1* mutations exhibited more severe anemia with a significantly increased proportion of bone marrow (BM) early stage erythroblasts and reduced enucleated erythrocytes compared to CMML patients with WT *ASXL1*. Knockdown of *ASXL1* in cord blood CD34^+^ cells reduced erythropoiesis and impaired erythrocyte enucleation. Consistently, the BM and spleens of *VavCre*^+^;*Asxl1*^*f/f*^ (*Asxl1*^*∆/∆*^) mice had less numbers of erythroid progenitors than *Asxl1*^*f/f*^ controls. *Asxl1*^*∆/∆*^ mice also had an increased percentage of erythroblasts and a reduced erythrocyte enucleation in their BM compared to littermate controls. Furthermore, *Asxl1*^*∆/∆*^ erythroblasts revealed altered expression of genes involved in erythroid development and homeostasis, which was associated with lower levels of H3K27me3 and H3K4me3. Our study unveils a key role for ASXL1 in erythropoiesis and indicates that *ASXL1* loss hinders erythroid development/maturation, which could be of prognostic value for MDS/MPN patients.

Anemia is a major clinical feature of myelodysplastic syndrome (MDS), which is caused by ineffective erythropoiesis. Anemia is a prognostic factor in both the WHO Prognostic Scoring System (WPSS) and the Revised International Prognostic Scoring System (IPSS-R) for MDS^1^,^2^,^3^. In addition, anemia has been identified as an independent predictor of poor prognosis using multiple prognostic scoring systems for chronic myelomonocytic leukemia (CMML)^4^,^5^, including MD Anderson Prognostic Score (MDAPS), CMML-specific prognostic scoring system (CPSS), and Mayo prognostic model. Genomic studies have highlighted that *ASXL1* are recurrently mutated in spectra of myeloid malignancies, including MDS (~16%), CMML (~45%), and acute myeloid leukemia (AML, ~37%)^6^. Multiple clinical investigations indicate that *ASXL1* mutation is an independent poor prognostic marker in patients with MDS and CMML^6^,^7^,^8^,^9^,^10^. However, the role of *ASXL1* in normal erythropoiesis and the effect of *ASXL1* mutations on ineffective erythropoiesis in patients with MDS or CMML remain to be elucidated.

While transcriptome changes during erythropoiesis are well documented^11^,^12^, the mechanisms concerning chromatin remodeling remain unclear. Dysregulated histone modifications have been shown to modulate gene activation, gene silencing, or a bivalent state in many hematologic disorders, including severe anemia, myelodysplastic disorders, and leukemia^13^. A recent study using CD34^+^ cell-derived erythroblasts suggests lineage- and developmental stage-specific regulation by polycomb repressive complex 2 (PRC2)^14^. In addition, ASXL1 regulates epigenetic marks and transcription through interaction with PRC2 and other transcriptional activators and repressors^15^,^16^,^17^.

We have reported that *Asxl1*^*−/−*^ and adult *Asxl1*^+/*−*^ mice develop anemia^18^,^19^. In the current study, we show that knockdown of *ASXL1* in cord blood (CB) CD34^+^ cells impairs erythropoiesis and results in decreased erythrocyte enucleation. *Asxl1* deletion in *VavCre*^+^;*Asxl1*^*f/f*^ (*Asxl1*^*∆/∆*^) mice reduced erythroid progenitors in the bone marrow (BM) and spleens, and hindered erythroid terminal differentiation. Furthermore, *Asxl1*^*∆/∆*^ erythroblasts had decreased levels of global histone H3 lysine 27 trimethylation (H3K27me3) and lysine 4 trimethylation (H3K4me3), which are associated with the altered expression of genes involved in erythroid development and homeostasis. Therefore, our study defines a key role for ASXL1 in erythropoiesis and shows that *ASXL1* (*Asxl1*) loss hinders erythroid development and terminal maturation. This study identifies an additional biological function of ASXL1 and shows that the identification of mutations in *ASXL1* could be of prognostic value for CMML patients.

## Results

### More severe anemia in MDS/MPN patients with *ASXL1* mutations

**Figure 1:**
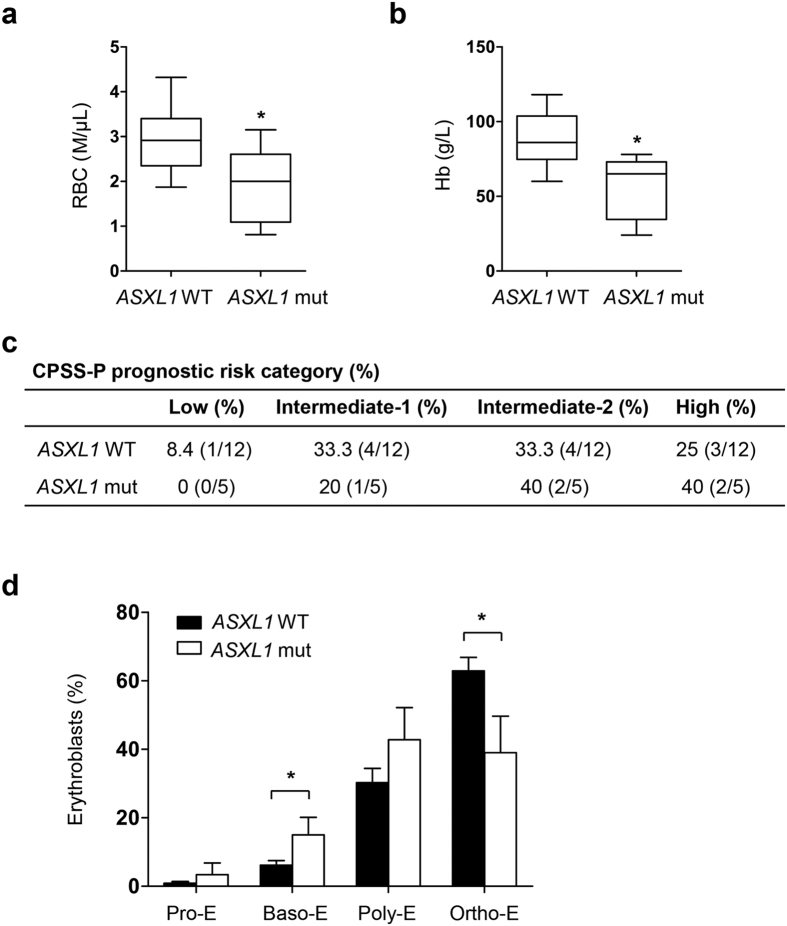
Severe erythroid phenotype in MDS/MPN patients with *ASXL1* mutation. (**a,b**) RBC counts (**a**) and hemoglobin levels (**b**) in MDS/MPN patients with WT *ASXL1* (n = 12) and MDS/MPN patients with *ASXL1* mutations (n = 6). (**c**) CPSS-P prognostic risk category of the 17 patients with CMML. Modified CMML-specific prognostic scoring system including platelet count (CPSS-P) was used for prognostic evaluation. CPSS-P divided the patients into four risk groups (low, intermediate-1, intermediate-2, and high) based on their CMML FAB type, CMML WHO type, CMML-specific cytogenetics, RBC transfusion dependence and platelet count. (**d**) The percentage of Pro-E, Baso-E, Poly-E and Ortho-E populations in MDS/MPN patients with (n = 6) or without (n = 12) *ASXL1* mutations in the BM. Compared to patients with WT *ASXL1*, patients with *ASXL1* mutations had an increased proportion of Baso-E and a decreased proportion of Ortho-E. Data are presented as mean ± SEM. *p < 0.05.

Abnormal erythropoiesis in the BM is a key characteristic in patients with MDS or MDS and myeloproliferative neoplasms (MPN) overlapping diseases (MDS/MPN). We compared the erythroid parameters in MDS/MPN patients with (n = 6) or without (n = 12) *ASXL1* mutations (Supplementary Fig. S1a). The patients with *ASXL1* mutations had lower red blood cell (RBC) counts and hemoglobin (Hb) levels compared to those with WT *ASXL1* (Fig. 1a,b). Based on a modified CMML-specific prognostic scoring system including platelet count (CPSS-P), 17 patients with CMML were segregated into four prognostic categories for overall survival (OS): low-, intermediate-1, intermediate-2, and high-risk categories. A total of 40% of the patients with *ASXL1* mutations were in the intermediate-2 category and 40% of patients were in the high-risk category, while 33.3% and 25% of the patients without *ASXL1* mutations were in the intermediate-2 category and high-risk category, respectively (Fig. 1c)^5^. The Mayo prognostic scoring model validated the results in the same cohort of patients (Supplementary Fig. S1b)^20^. In addition, a significantly higher percentage of basophilic erythroblasts (Baso-E) was observed in the BM of patients with *ASXL1* mutations compared to the patients with WT *ASXL1* (Fig. 1d). Although not significant, a trend toward an increase in polychromatic erythroblasts (Poly-E) was observed in patients with *ASXL1* mutations compared to those with WT *ASXL1*. In contrast, a decreased proportion of orthochromatic erythroblasts (Ortho-E) was observed in the BM of MDS/MPN patients with *ASXL1* mutations (Fig. 1d). Blood smear analysis revealed a relatively higher frequency of erythroid cells with dysplastic features in a CMML patient with an *ASXL1* mutation (c.1772dupA; p.Y591X) compared to the patients with WT *ASXL1* (Supplementary Fig. S1c,d). These data demonstrate that ASXL1 plays an important role in erythroid development and the terminal maturation of erythrocytes.

### Knockdown of *ASXL1* in CB CD34^+^ cells impairs erythroid differentiation and decreases the number of enucleated erythrocytes

**Figure 2:**
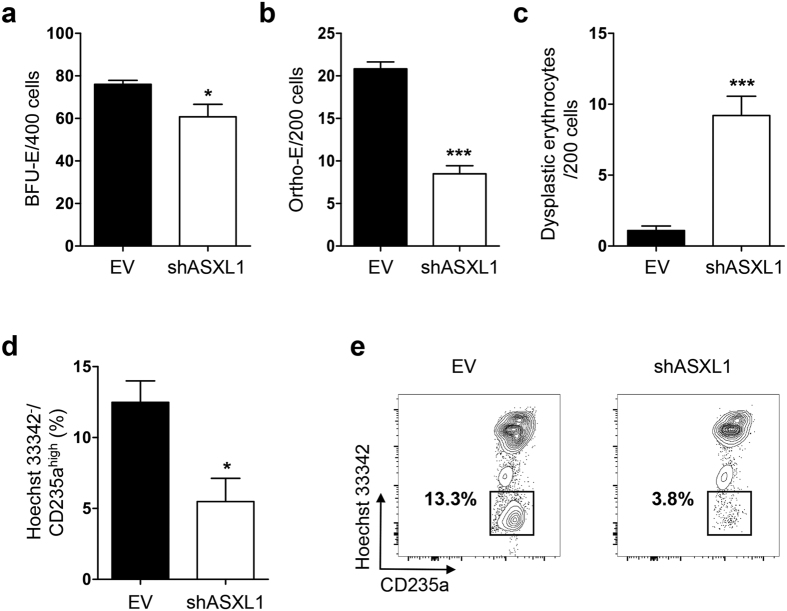
Knockdown of *ASXL1* in CB CD34^+^ cells impairs their erythroid differentiation. (**a**) CB CD34^+^ cells were transduced with lentivirus carrying shASXL1 or empty vector (EV) for 2 days. The sorted GFP^+^ cells were cultured in methylcellulose for 14 days. The numbers of BFU-E from CB CD34^+^ cells transduced with shASXL1 were significantly lower than those from EV controls. Data are represented as mean ± SEM from three independent experiments. *p < 0.05. **(b,c)** May-Giemsa stained cytospin preparation from the cultures of GFP^+^ CD34^+^ cells expressing shASXL1 or EV were analyzed on day 12. A quantitative comparison based on the cell counting of different erythroid progenitors and dyserythropoietic cells in 200 cells/field from 10 different fields revealed that cells transduced with shASXL1 contained a lower percentage of Ortho-E (**b**) and a higher percentage of dyserythropoietic cells (**c**) compared to the EV. Data are represented as mean ± SEM from three independent experiments. ***p *<* 0.001. **(d,e)** Analysis of erythrocyte enucleation after ASXL1 knockdown. After 10 days of culture in methylcellulose, 5 individual colonies were isolated from ASXL1 knockdown or EV CD34^+^ cells and seeded in IMDM basic medium supplemented with EPO. After an additional 6 days, the cells were stained with Hoechst 33342, and anti-CD235a antibodies. The percent of Hoechst 33342^−^/CD235a^high^ enucleated erythrocytes were determined. *ASXL1-KD* cultures contained a decreased proportion of enucleated erythrocytes compared to the EV cultures. Data are represented as mean ± SEM from three independent experiments. *p < 0.05.

To determine whether *ASXL1* loss affects erythropoiesis, we transduced *ASXL1* shRNA (shASXL1) into CB CD34^+^ cells (Supplementary Fig. S2a) to achieve *ASXL1* knockdown (KD) and examined erythroid development by burst forming unit-erythroid (BFU-E) assays, flow cytometric analysis and morphologic analysis of cytospin preparations. The GFP^+^ cells were sorted and plated in semi-solid methylcellulose as well as in liquid cultures (Supplementary Fig. S2b). The lentivirus carrying shASXL1 led to a ~50% downregulation of *ASXL1*, and the *ASXL1* levels remained approximately 50% throughout the entire period of erythroid differentiation (Supplementary Fig. S2c). *ASXL1*-KD CB CD34^+^ cells gave rise to a significant lower frequency of BFU-E compared to the cells transduced with empty vector (EV) control (Fig. 2a). Compared to EV control, the transduction of shASXL1 resulted in a significant reduction in Ortho-E in CB CD34^+^ cells (Fig. 2b) and an increased proportion of dysplastic erythrocytes (Fig. 2c), including budding, internuclear bridging, karyorrhexis, multinuclearity and megaloblastoid changes (Supplementary Fig. S2d). Hoechst 33342 staining demonstrated a significant reduction in the Hoechst 33342^−^/CD235a^high^ cell population in *ASXL1*-KD cultures compared to EV control (Fig. 2d,e), suggesting impaired enucleation of erythroblasts from *ASXL1*-KD CD34^+^ cells.

### *Asxl1* loss impairs erythroid development and maturation

**Figure 3:**
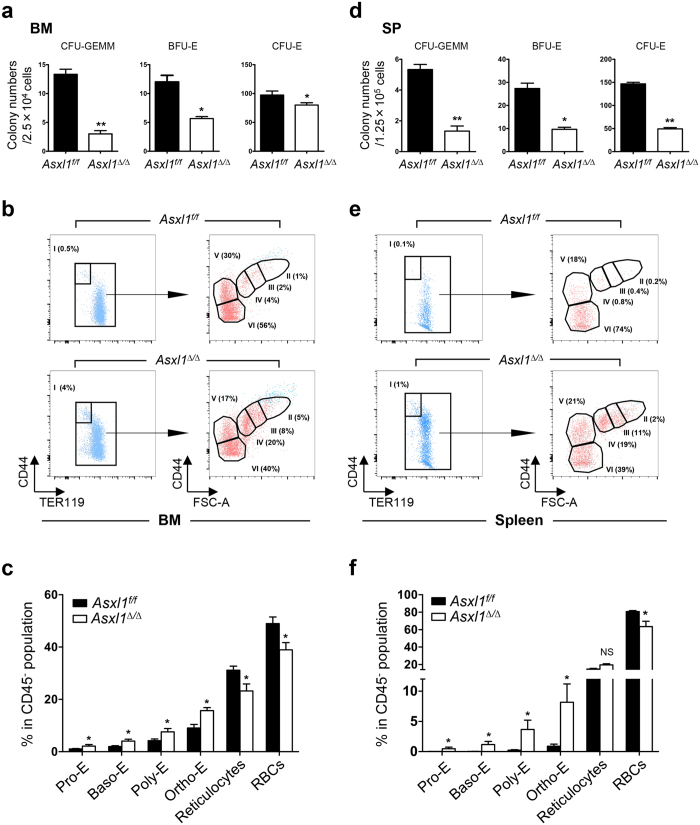
Loss of *Asxl1* impairs erythroid terminal maturation. (**a,d**) Colony forming unit (CFU) assays with BM cells and spleen (SP) cells from *Asxl1*^∆/∆^ and *Asxl1*^*f/f*^ mice (3 mice/genotype from two independent experiments). (**b,e**) Representative flow cytometry dot plots show the percentage of various erythroid subsets in the BM (**b**) and SP (**e**) of *Asxl1*^*f/f*^ and *Asxl1*^∆/∆^ mice. Dot plots in (**b,e**) show CD45^−^Mac1^−^Gr.1^−^ gated cells. Pro-E (CD44^hi^TER119^low^, population I), Baso-E (population II), Poly-E (population III), Ortho-E (population IV), reticulocytes (population V), and red blood cells (population VI) groups were identified according to their forward scatter (FSC) and CD44 expression. (**c,f**) Quantitation of the erythroid populations in the BM (**c**) and SP (**f**) of *Asxl1*^*f/f*^ and *Asxl1*^∆/∆^ mice (n = 5 mice/genotype). Data were presented as mean ± SEM. *p < 0.05, **p < 0.01.

Erythroblasts are derived from hematopoietic stem/progenitor cells (HSPCs). Erythroid progenitors give rise to lineage-restricted erythroid progenitors, erythroblasts, and ultimately erythrocytes. The earliest erythroid progenitor cells (BFU-E) are the first cell-committed progenitors, which give rise to later progenitors known as colony forming unit-erythroid cells (CFU-E) that undergo terminal differentiation to enucleated RBCs^21^. We have previously shown that global deletion of *Asxl1*, or polyinosinic:polycytidylic ribonucleic acid (pI:pC) injection mediated *Asxl1* loss in mice leads to decreased RBC counts and lower Hb levels^18^,^19^. Here, using *VavCre* transgenic mice to drive *Asxl1* deletion in hematopoietic system (*Asxl1*^*∆/∆*^), we reported that the erythroid parameters of peripheral blood (PB), including RBC counts, Hb levels and percentage of hematocrit (HCT) were significantly lower in *Asxl1*^*∆/∆*^ mice than that in *VavCre*^*−*^*;Asxl1*^*f/f*^ (*Asxl1*^*f/f*^) littermates (Supplementary Fig. S3a), reinforcing the important intrinsic role of *Asxl1* in erythropoiesis *in vivo*. To determine the cellular mechanism underlying the lower RBC counts and Hb levels in *Asxl1*^∆/∆^ mice, we assayed colony forming unit-granulocyte/erythrocyte/monocyte/megakaryocyte (CFU-GEMM), BFU-E and CFU-E using BM and spleen cells from *Asxl1*^∆/∆^ mice. Significantly decreased numbers of CFU-GEMM, BFU-E and CFU-E were observed in both the BM and spleen cells of *Asxl1*^∆/∆^ mice compared to littermate control mice (Fig. 3a,d). These data suggest that loss of *Asxl1* decreases the frequencies of erythroid progenitors and impairs erythroid maturation *in vivo*.

The CFU-E cells develop through a series of maturational stages, including proerythroblasts (Pro-E), Baso-E, Poly-E, and eventually Ortho-E, reticulocytes and mature RBCs^22^,^23^. During erythrocyte maturation, TER119^+^ cells lose CD44 expression and decrease their size^24^. The evaluation of TER119/CD44 expression by flow cytometric analysis can phenotypically distinguish the developmental stages of erythroid cells. To determine the specific stage(s) of erythroid differentiation that is affected upon *Asxl1* loss *in vivo*, we next examined the percentages of cells in different stages of the erythroid lineage using flow cytometric analysis following the staining of BM and spleen cells with antibodies against CD45, Gr1, Mac1, CD44, and TER119 (Supplementary Fig. S3b), as previously reported^23^,^25^,^26^. Significantly higher percentages of Pro-E (I), Baso-E (II), Poly-E (III) and Ortho-E (IV) populations were observed in the BM (Fig. 3b,c) and spleens (Fig. 3e,f) of *Asxl1*^∆/∆^ mice compared to *Asxl1*^*f/f*^ control mice. In contrast, a marked reduction of RBCs (VI) was observed in the BM (Fig. 3b,c) and spleens (Fig. 3e,f) of *Asxl1*^∆/∆^ mice compared to *Asxl1*^*f/f*^ control mice. Within the nucleated erythroblast population, the Pro:Baso:Poly:Ortho ratio in the BM of control mice followed a 1:2:4:8 pattern^24^. In contrast, the Pro:Baso:Poly:Ortho ratio in the BM of *Asxl1*^∆/∆^ mice was 1:1.25:2:5 (Fig. 3b). Similarly, the ratio of Pro:Baso:Poly:Ortho erythroblasts in the *Asxl1*^∆/∆^ spleens was 1:2:11:19 compared to a ratio of 1:2:4:8 in *Asxl1*^*f/f*^ spleens (Fig. 3e), demonstrating abnormal erythroid development in *Asxl1*^∆/∆^ mice *in vivo*.

Morphologic analysis of BM cell cytospin in *Asxl1*^+/*−*^ mice revealed dysplastic features of erythroblasts, including karyorrhexis, nuclear budding, mitotic erythroblast, binucleated erythrocytes and nuclear bridging (Supplementary Fig. S3c, I-IV, respectively). Collectively, these data indicate that ASXL1 plays an important role in erythroid development and terminal maturation.

### Loss of *Asxl1* impairs erythrocyte enucleation and increases apoptosis *in vivo*

**Figure 4:**
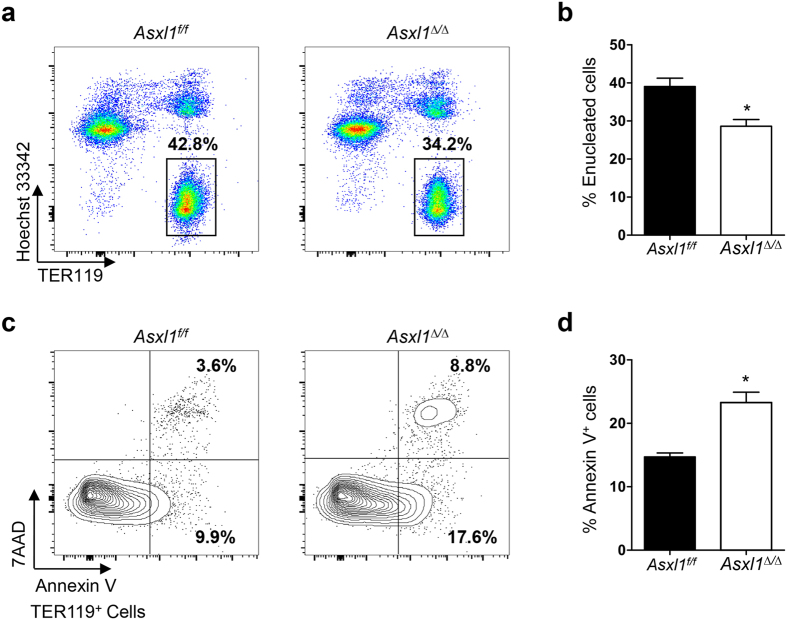
Loss of *Asxl1* impairs enucleation and increases the apoptosis of erythroid cells *in vivo*. BM cells from *Asxl1*^*f/f*^ and *Asxl1*^∆/∆^ mice were stained with TER119/Hoechst 33342 or 7AAD/Annexin V and flow cytometric analysis was performed to quantify erythroid enucleation or apoptosis. (**a**) Representative dot plots show the percentage of erythroid enucleation in the BM of *Asxl1*^*f/f*^ and *Asxl1*^∆/∆^ mice. (**b**) Quantification of enucleated erythroid cells shown in (a) (TER119^+^ and Hoechst 33342^−^, n = 5 mice/genotype). (**c**) Representative contour plots of apoptosis show early and late apoptotic cell populations (7AAD^−^Annexin V^+^ and 7AAD^+^ Annexin V^+^). (**d)** Quantification of Annexin V-positive cells shown in (**c**) (n = 3 mice/genotype). Data were presented as mean ± SEM. *p < 0.05.

The enucleation of erythrocytes is a critical step in the terminal differentiation of red blood cells. During the enucleation of erythroblast, the Hoechst 33342-positive nucleated cells turn into Hoechst 33342-negative enucleated cells. To determine whether the reduced erythrocyte maturation in *Asxl1*^∆/∆^ mice is associated with alterations in erythroblast enucleation, we next performed flow cytometric analysis following Hoechst 33342 and TER119 staining and examined the extent of enucleation in *Asxl1*^∆/∆^ BM cells. A significantly lower percentage of Hoechst 33342^−^/TER119^+^ cells was observed in the BM of *Asxl1*^∆/∆^ mice compared to *Asxl1*^*f/f*^ control mice (Fig. 4a,b), suggesting the impaired enucleation of erythroblasts in *Asxl1*^∆/∆^ mice *in vivo*.

Definitive erythropoiesis in embryos occurs in the fetal liver in mice. To verify the important role of *Asxl1* in erythroid development, the fetal livers of WT, *Asxl1*^+/*−*^ and *Asxl1*^*−/−*^ embryos on embryonic day 13.5 were processed for cytospin followed by May-Giemsa and benzidine counter staining. Quantitative analysis of the cytospins revealed a marked reduction of benzidine^+^ erythrocytes in both *Asxl1*^+/*−*^ and *Asxl1*^*−/−*^ embryos (Supplementary Fig. S4a,b), verifying a role of *Asxl1* in maturation in mammalian erythroblasts. In addition, the impaired maturation was gene dosage-dependent (Supplementary Fig. S4a,b). During erythrocyte maturation in the fetal liver, the evaluation of TER119/CD71 expression by flow cytometric analysis can phenotypically identify distinct stages of erythroid cells, as previously reported (Supplementary Fig. S4c)^27^. We next examined the percentage of different stages of erythroid lineage cells with flow cytometric analysis following the staining of fetal liver cells with antibodies against CD71 and TER119. Significantly higher percentages in the populations of S2 cells were observed in the fetal livers (Supplementary Fig. S4d) of *Asxl1*^+/*−*^ and *Asxl1*^*−/−*^ mice compared to WT control mice. In contrast, a reduced number of S4 and S5 cells was observed in the fetal livers of *Asxl1*^+/*−*^ and *Asxl1*^*−/−*^ mice compared to WT control mice. These results suggest a cell-autonomous effect of *Asxl1* on erythroid maturation.

We previously reported that *Asxl1* loss increased the apoptosis of myeloid cells^18^. To examine whether the loss of *Asxl1* also affects the apoptosis of erythrocytes, we performed flow cytometric analysis on the BM cells of *Asxl1*^*∆/∆*^ mice following Annexin V/7AAD and TER119 antibody staining. The proportion of Annexin V^+^ cells in TER119^+^ erythroblasts was significantly increased in the BM of *Asxl1*^∆/∆^ mice compared to *Asxl1*^*f/f*^ control mice (Fig. 4c,d). Consistently, May-Giemsa–stained PB smear revealed apoptotic and dysplastic features in the erythrocytes of *Asxl1*^∆/∆^ mice (Supplementary Fig. S4e). Collectively, these data indicate that *Asxl1* loss-mediated anemia is a consequence of the combinatorial effects of impaired erythroid maturation and increased apoptosis.

### Deletion of *Asxl1* altered the expression of genes important for erythropoiesis and reduced the global levels of H3K27me3 and H3K4me3 in TER119^+^ cells

**Figure 5:**
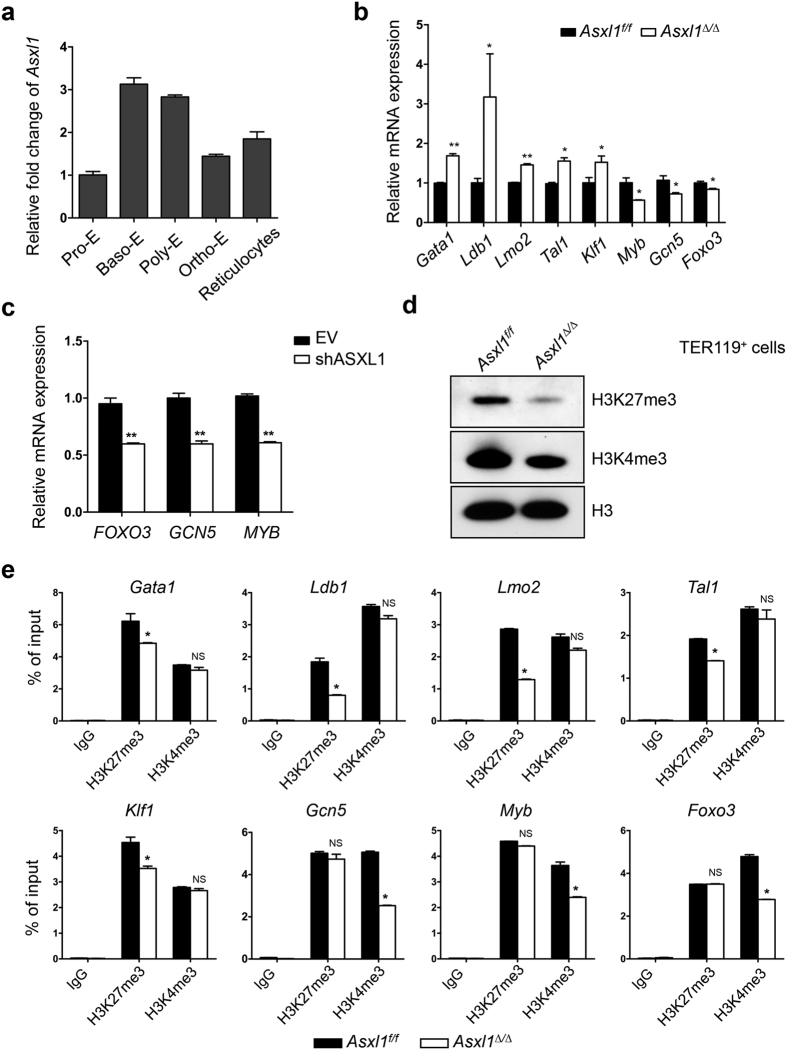
Deletion of *Asxl1* dysregulates genes critical for erythropoiesis through altered H3K27me3 and H3K4me3 occupancy in TER119^+^ cells. (**a**) Relative expression level of *Asxl1* in purified erythroblast populations. (**b**) qPCR analysis of critical genes associated with erythroid differentiation and maturation in TER119^+^ cells from *Asxl1*^∆/∆^ and *Asxl1*^*f/f*^ (n = 3 mice/genotype). Data are shown as expression units relative to the respective gene expression in *Asxl1*^*f/f*^ mice using β-actin as an internal calibrator. (**c**) The mRNA expression of *FOXO3*, *GCN5*, and *MYB* in the erythroid progenitors of CB CD34^+^ cells transduced with shASXL1 after 12 days of culture *in vitro*. Data are shown as expression units relative to the respective gene expression in control cells, using *18S* as an internal calibrator. (**d**) Western blot analysis of H3K27me3 and H3K4me3 in the TER119^+^ cells from *Asxl1*^∆/∆^ and *Asxl1*^*f/f*^ mice. The total H3 levels served as a loading control. Representative blots from 3 independent experiments are shown. (**e**) ChIP-qPCR for H3K27me3 and H3K4me3 at/around the *Gata1, Ldb1, Lmo2, Tal1, Klf1*, *Myb, Gcn5*, and *Foxo3* TSS regions (n = 3 mice/genotype from two independent experiments). Data are represented as mean ± SEM. *p < 0.05, **p < 0.01.

To determine *Asxl1* expression levels throughout erythrocyte differentiation, quantitative PCR (qPCR) was performed using RNA extracted from a purified sample of various erythroid cell populations. As shown in Fig. 5a, *Asxl1* is expressed throughout erythroblast differentiation. The expression levels of *Asxl1* in Baso-E and Poly-E were higher than in Pro-E, suggesting a role for ASXL1 during erythroid development and maturation. The specification and differentiation of erythroid cells have been well studied and are regulated at the transcriptional, posttranscriptional, and epigenetic levels. ASXL1 regulates gene transcription through interaction with PRC2 and other transcription activators and repressors^15^,^16^,^17^. To determine whether *Asxl1* loss alters the expression of genes that are critical for erythropoiesis, qPCR was performed using TER119^+^ cells of *Asxl1*^*f/f*^ and *Asxl1*^*∆/∆*^ mice. The expression of *Gata1*, *Ldb1*, *Lmo2*, *Tal1*, and *Klf1* was up-regulated and the expression of *Foxo3, Myb*, and *Gcn5* was down-regulated in *Asxl1*^∆/∆^ TER119^+^ erythroblasts (Fig. 5b). Consistently, the expression of *FOXO3*, *GCN5* and *MYB* was also downregulated in CD235a^+^ cells differentiated from shASXL1 CB CD34^+^ cells (Fig. 5c).

ASXL1 recruits PRC2 to the chromatin and *Asxl1* depletion in myeloid progenitor cells leads to global reduction of H3K27me3^18^,^28^ and H3K4me3^18^, hallmark repressive and active H3 modifications, respectively. Interestingly, both H3K27me3 and H3K4me3 were reduced in *Asxl1*^*∆/∆*^ TER119^+^ cells compared to *Asxl1*^*f/f*^ TER119^+^ cells (Fig. 5d). However, deletion of *Asxl1* did not affect the H2AK119ub1 levels in TER119^+^ nucleated cells compared to *Asxl1*^*f/f*^ cells (data not shown). To examine whether the altered expression of these genes is associated with changes of H3K27me3 and H3K4me3 in their transcription start site (TSS), ChIP-qPCR was performed using antibodies against either H3K27me3 or H3K4me3. ChIP-qPCR showed reduced H3K27me3 enrichment at/around the transcription start site of each of the up-regulated genes (*Gata1*, *Ldb1*, *Lmo2*, *Tal1*, *Klf1*) and reduced H3K4me3 enrichment at/around the transcription start site of each of the down-regulated genes (*Foxo3, Myb, Gcn5*) (Fig. 5e). These results indicate that ASXL1 is required to maintain the H3 methylation states of important genes in erythroblasts, thereby controlling normal expression of genes during erythropoiesis.

## Discussion

Although a high frequency of *ASXL1* mutations is found in MDS and CMML, the role of *ASXL1* in erythropoiesis and the effect of *ASXL1* mutations on defective erythropoiesis remain to be explored. Here, we report that knockdown of *ASXL1* in CB CD34^+^ cells reduced the BFU-E frequency and impaired erythroid terminal differentiation. Importantly, MDS/MPN patients with *ASXL1* mutations have more severe anemia and higher proportions of early-stage erythroblasts compared to patients with WT *ASXL1*. Therefore, *ASXL1* mutations may impair erythroid commitment and terminal differentiation, contributing to the refractory anemias experienced by MDS and MDS/MPN patients with *ASXL1* mutations.

To improve our understanding of the role of ASXL1 in erythropoiesis, we performed detailed phenotypic analyses of erythropoiesis using our newly developed *Asxl1*-deficient murine models. Our results show that *Asxl1*-deficient mice exhibited anemia with impaired erythroid commitment and terminal erythroid maturation. We previously reported that global deletion of *Asxl1* resulted in a significant reduction of CFU-GEMM and BFU-E^18^. To define the cell-autonomous effect of *Asxl1* loss in erythropoiesis, we used *VavCre^+^;Asxl1*^*f/f*^ (*Asxl1*^∆/∆^) mice for colony-forming unit assays to determine the frequency of erythroid progenitors in the BM and spleen. We found a significant reduction in the frequencies of BFU-E and CFU-E in the BM and spleen cells of *Asxl1*^*∆/∆*^ mice compared to littermate controls, reinforcing the cell intrinsic effect of ASXL1 in erythropoiesis. Erythropoiesis is a dynamic process starting from multipotent hematopoietic progenitor cells that differentiate into mature enucleated red blood cells. During erythrocyte development, erythroblasts can be classified as pro-, basophilic, polychromatophilic, or orthochromatic erythroblasts. We found that the loss of *Asxl1* reduced the enucleation of erythroblasts, increased apoptosis and induced dysplastic features in the erythroid lineage *in vivo*. It is likely that the accumulation of each of the erythroblast subtypes and decreased numbers of reticulocytes/red blood cells are the result of the combinatorial effects of impaired enucleation and apoptosis in *Asxl1*^*∆/∆*^ mice. This result was further validated by *in vitro* erythroid cultures of BM and fetal liver cells. Our study suggests a cell-autonomous effect of ASXL1 in erythropoiesis.

Erythrocyte development requires the coordinated expression and activity of several transcription factors for the induction of erythroid-specific genes^21^. Among these, *Gata1*, *Tal1*, and *Klf1* have been shown to be critical, earning the designation of erythroid master regulators. *Asxl1* loss in both cKit^+^ and TER119^+^ cells results in the dysregulated expression of multiple regulatory factors, including the upregulation of *Ldb1* and *Lmo2*, and the downregulation of *Foxo3*, *Myb* and *Gcn5*. It has been reported that the overexpression of GATA1 in erythroid cells inhibits their differentiation, leading to lethal anemia^29^. Upregulated *Gata1* gene expression in *Asxl1*-null mice contributes to the impairment in erythroid progenitor differentiation. A recent study showed that FOXO3 functions as a critical determinant of the erythroid cell transcriptome, cooperating with these factors and their requisite co-regulators to control specific molecular/cellular steps that drive terminal erythroid maturation^30^. The decreased expression of *Foxo3* may also contribute to the impaired erythroid differentiation in *Asxl1*-null mice. *Foxo3* has also been shown to be essential for erythroblast enucleation^30^. The decreased *Foxo3* expression, together with dysregulated erythroid regulatory factors, such as *Ldb1*, *Lmo2*, *Tal1*, and *Klf1* may lead to the defective terminal maturation of erythrocytes.

ASXL1, one of the mammalian Asx homologs, directly interacts with PRC2. We and others have previously reported that *Asxl1* loss results in a reduction in global levels of H3K27me3, the repressive histone methylation mark regulated by the PRC2 complex, in hematopoietic stem/progenitor cells^18^,^19^. Our results show that *Asxl1* loss in the TER119^+^ population also diminishes the global levels of histone H3K27me3 and H3K4me3. *Asxl1* is enriched at promoter regions genome wide^19^, suggesting a potential role for ASXL1 in the direct regulation of gene transcription. Our ChIP-qPCR results confirmed that the dysregulation of these key regulatory factors for erythropoiesis is associated with the altered occupancy of histone H3K27me3 and H3K4me3 in the TSS regions of these genes.

Collectively, *ASXL1* (*Asxl1*) loss impairs erythroid development and hinders erythroid differentiation. Our study provides new insight into the key role of ASXL1 in normal erythropoiesis. This study leads to the identification of additional biological function of ASXL1 with prognostic value for MDS and MDS/MPN patients.

## Methods

### Mouse Models

The generation of *Asxl1:nlacZ/nGFP* knock-in and *Asxl1*^*f/f*^ mice has been previously described^18^,^19^. *VavCre* transgenic mice were purchased from Jackson Laboratories. All of the animal models were bred on a C57BL/6 genetic background. All animal experiments were conducted in accordance with the Guidelines for the Care and Use of Laboratory Animals. All animal protocols were approved by the University of Miami Institutional Animal Care and Use Committee.

### Human subjects

Physical examinations and medical histories were obtained for a cohort of 18 individuals with MDS/MPN from the Institute of Hematology and Blood Diseases Hospital, Chinese Academy of Medical Sciences & Peking Union Medical College. Written informed consent was obtained from all patients and the study was approved by the Ethics Committee at the Institute of Hematology & Blood Diseases Hospital, Chinese Academy of Medical Sciences according to guidelines of the 1975 Helsinki Declaration. The diagnosis of MDS/MPN was based on the 2008 WHO diagnostic criteria. Human CB CD34^+^ cells were isolated from the cord blood of healthy donors after obtaining informed consent. The percentages of pro-erythroblasts, basophilic erythroblasts, polychromatic erythroblasts and orthochromatic erythroblasts were calculated based on erythroblast cell counting of samples from the BM of MDS/MPN patients with or without *ASXL1* mutations.

### Clonogenic assays

Colony-forming units in cultures (CFU-Cs) were assayed as previously described^31^. Briefly, single-cell suspensions of mononuclear cells from the BM and spleen were seeded onto a 35-mm gridded dish containing Methocult (StemCell Technologies, Vancouver, Canada) and incubated at 37 °C in a humidified atmosphere containing 5% CO_2_ in air^18^. All experiments were performed in triplicate. The colonies were scored on day 2 for CFU-E and day 7 for BFU-E and CFU-GEMM.

### Transduction of CB CD34^+^ cells and erythroid differentiation

Human CB CD34^+^ cells were enriched by positive selection using a magnetic bead sorting system, according to the manufacturer’s instructions (Miltenyi Biotec, Bergisch Gladbach, Germany). For the initial expansion, CB CD34^+^ cells were cultivated in StemSpan SFEM serum-free medium (StemCell Technologies) supplemented with 100 ng/mL human stem cell factor (SCF), 100 ng/mL Fms-like tyrosine kinase 3 ligand (Flt-3L) and 50 ng/mL thrombopoietin (TPO) for 2 days^32^. The pre-stimulated CB CD34^+^ cells were then transferred on day 0 into erythroid differentiation culture medium (IMDM basic culture medium supplemented with 10^−6^ M hydrocortisone (Sigma, St Louis, USA), 100 ng/mL SCF, 3 IU/mL erythropoietin (EPO) and 5 ng/mL Interleukin-3 (IL-3, Peprotech, Rocky Hill, NJ), and infected with lentivirus carrying shRNA targeting *ASXL1* or an empty vector control in the presence of polybrene (5 μg/mL, Sigma). GFP^+^ cells were sorted on the flow cytometer (FACS Aria III, BD Biosciences, San Jose, CA) 48 hours post-infection, and used for the following experiments. For BFU-E assays, the sorted GFP^+^ cells were cultured in methylcellulose (H4434, StemCell Technologies) supplemented with 10% IMDM (Sigma) basic culture medium for 14 days. The left GFP^+^ cells were continued to culture under the same liquid culture condition for an additional 7 days until Day 9 (Supplementary Fig. S2b). On day 9, the cells were resuspended at 3 × 10^5^/mL in fresh medium with an additional 100 ng/mL SCF and 3 IU/mL EPO. On day 15, the cells were cultured at concentration of 10^6^/mL in the presence of only 3 IU/mL EPO at 37 °C with 5% CO_2_^33^,^34^,^35^. On day 9, 12, 15, and 18, the GFP^+^ cells were sorted again to determine the knockdown efficiency of *ASXL1* by qPCR. To determine the cells’ morphologic changes, GFP^+^ cells (1 × 10^5^ cells/mL) were also processed for cytospin on poly-L-lysine-coated slides and were processed for May-Giemsa staining. The cells were imaged and cell morphology was analyzed using phase contrast microscopy (Nikon, Tokyo, Japan). Erythroid clones generated in semisolid culture after 10 days were individually isolated and seeded in IMDM basic culture medium in the presence of 3 IU/mL EPO. Six days later, the cells were collected and stained with antibodies against human CD71 (APC-Cy7, BioLegend, San Diego, CA), CD235a (PE, eBioscience, San Diego, CA) and Hoechst 33342 (5 mg/mL, Sigma) to determine the enucleation of erythroblast.

### Flow cytometric analysis

The cells from BM, spleen, and fetal liver were passed through a 23-G needle and filtered through a 70-μM cell strainer to obtain single-cell suspensions. The cells were stained with antibodies, including CD71 (FITC), TER119 (PE), CD44 (APC), CD45 (PE-Cy7), Gr1 (PE-Cy7), CD11b (PE-Cy7). All of the antibodies were purchased from BD Pharmingen. Following incubation of cells with conjugated antibodies for 30 minutes at 4 °C, cells were washed in PBS containing 0.5% bovine serum albumin (BSA) and analyzed using a FACS Calibur cell analyzer (BD Biosciences). For enucleation analysis, BM cells from *Asxl1*^*f/f*^ and *Asxl1*^*∆/∆*^ mice were stained with TER119-PE (BD Pharmingen) for 30 mins at room temperature and nuclear staining was performed with 10 μg/mL Hoechst 33342. Data were acquired on FACS LSR II instrument (BD Biosciences).

### Apoptosis assay

BM cells of *Asxl1*^*f/f*^ and *Asxl1*^*∆/∆*^ mice were staining with TER119 (PE) for 30 mins. Apoptosis analysis was performed using a PE-Annexin V Apoptosis Detection kit (BD Pharmingen). In brief, 5 μL of Annexin V and 5 μL of 7-amino-actinomycin (7-ADD) were added in 100 μL suspended cells stained by TER119, and the mixture was incubated at room temperature for 25 min. Then 400 μL of binding buffer was added and the extent of apoptosis and staining pattern of the cells were tracked by flow cytometric analysis on a FACS LSR II instrument.

### Chromatin immunoprecipitation (ChIP)

TER119^+^ cells were fixed with 1% formaldehyde (Sigma) for 10 minutes at room temperature and quenched with 0.125 M glycine (Covaris Inc., Woburn, MA). The chromatin was isolated and sonicated into fragments with an average length of 200- to 300-bp. Genomic DNA regions of interest were isolated using antibody against H3K4me3 (ab8580, Abcam, Cambridge, UK) and H3K27me3 (ab6002, Abcam). A negative control was performed by incubating sonicated DNA with IgG. An aliquot of chromatin was precleared with protein A agarose beads (Invitrogen, Carlsbad, CA). Complexes were eluted from the beads and subjected to RNase and proteinase K (Qiagen, Valencia, CA) treatment. The crosslinks were reversed by incubation overnight at 65 °C, and ChIP DNA was purified by phenol-chloroform extraction and ethanol precipitation and was then analyzed by qPCR. The primers were designated to amplify 100-bp amplicons distributed along *Asxl1*^∆/∆^ mouse 5000-bp promoters (Table 1).

### Gene expression

Total RNA was extracted with TRIzol reagent (Invitrogen). Reverse transcription PCR amplifications were performed using M-MLV (Invitrogen) according to the manufacturer’s instructions. qPCR was performed in triplicate using an ABI 7500 with fast SYBR green master mix (Applied Biosystems, Waltham, MA). *β-actin*, *GAPDH* and 18S were used as the reference gene and expression differences were calculated. Primers used are listed in Table 1.

### Western Blot Analysis

TER119^+^ cell lysates were subjected to western blot analysis^18^. Isolated proteins were fractionated using NuPAGE 4–12% Bis-Tris Gels (Invitrogen) and electro-transferred to PVDF membranes (Roche, Basel, Switzerland). Immunoblots were performed using specific antibodies. After incubation with anti-rabbit IgG or anti-mouse IgG (GE Healthcare, Piscataway, NJ) antibodies conjugated with HRP, the signals were detected using ECL chemiluminescence substrate (Thermo Fisher Scientific, Waltham, MA).

### Statistical analysis

Differences between experimental groups were determined by Student’s *t* test or analysis of variance (ANOVA) followed by Newman-Keuls multiple comparison tests as appropriate. *p* values of less than 0.05 were considered significant.

## Additional Information

**How to cite this article**: Shi, H. *et al*. ASXL1 plays an important role in erythropoiesis. *Sci. Rep*. **6**, 28789; doi: 10.1038/srep28789 (2016).

## Supplementary Material

Supplementary Information

## Figures and Tables

**Table 1:** Mouse and human primers, used for experimental procedures.

Gene Name	Forward	Reverse
mRNA(mouse)		
*Gata1*	GAGATCCTTGTGCCTCAGTTC	GGAGATGGTTGGGCTGTAAA
*Ldb1*	CTTAGTCCTCACCCTTGCCA	GCTTGAATGACTTTGAGGAACA
*Lmo2*	ATCGAAAGGAAGAGCCTGGA	TAGCGGTCCCCTATGTTCTG
*Tal1*	CACTGAGACTTGCCTTCCTATC	CTTGCTTTGCTTGCCCTAATG
*Klf1*	CCCATCACGTGAGTCTGAAAT	CTCGGAACCTGGAAAGTTTGTA
*Foxo3*	CTGAAGGATCACTGAGGAAAGG	CCACTTGCTGAGAGCAGATT
*Myb*	TGTGGCTGAGTTTCAAGAGAG	CCAGTGGTTTGAGCAGAAGA
*Gcn5*	TCTCACCTATGCTGACGAGTA	GTCCTTGATGTAGCCCAGATAG
mRNA(human)		
*ASXL1*	CCACAGCCCACTAAAGAGGA	CAGAGCACGGGCTTTAATGT
*GCN5*	CAGGGTGTGCTGAACTTTGTG	TCCAGTAGTTAAGGCAGAGCAA
*MYB*	CAGGAAGGTTATCTGCAGGAGT	CTATAGGCGGAGCCTGAGCAA
*FOXO3*	CTTCAAGGATAAGGGCGACA	AGTTCCCTCATTCTGGACCC
*GAPDH*	GAAGGTGAAGGTCGGAGTC	GAAGATGGTGATGGGATTTC
*h18S*	GCCTCAGTTCCGAAAACCA	ACCGCAGCTAGGAATAATGGA
ChIP(TSS)		
*Gata1*	ACCTGCAAAATGGGTACAGC	AGGCTATGTGTGGGTTGGAC
*Ldb1*	AGCCTGCAAGAGGAAAGGAT	ATTTTGCAAGGGGACACAAG
*Lmo2*	TTGATCCGGGCTGTTCTTAC	GGCCCGTTAGCTTATGATGA
*Tal1*	AACAGCCAGTTTCCACCATC	AGTGAGGGACAGAAGCCTGA
*Klf1*	GGCCAGCCTGGTCTACATAA	TGCGATATGTGTGTGGTGTG
*Foxo3*	TGTGAAGGTGCGATCTGAAG	AGCGGGCAACAGTTTTCTTA
*Myb*	CCGATCATCCCTCACACTCT	GGGTTGGGTGACACACTTCT
*Gcn5*	TGTCGGTGAACACTCTGCTC	ACTTGGTGGTGCGGATAGAC
