# Supplementary material for: ASXL1 plays an important role in erythropoiesis
Source: Sci Rep. 2016 Jun 29;6:28789. doi: 10.1038/srep28789 (PMC4926121; doi:10.1038/srep28789)
Supplement: Supplementary Information [file srep28789-s1.pdf]

Supplementary File

ASXL1 plays an important role in erythropoiesis

Hui Shi[1,2,3§], Shohei Yamamoto[1,2,4§], Mengyao Sheng[3§], Jie Bai[3], Peng Zhang[1,2], Runze Chen[1,2], Shi Chen[1,2], Lihong Shi[3], Omar Abdel-Wahab[5], Mingjiang Xu[1,2,], Yuan Zhou[3*] & Feng-Chun Yang[1,2*]

[1]Sylvester Comprehensive Cancer Center, [2]Department of Biochemistry and Molecular Biology, University of Miami Miller School of Medicine, Miami, FL 33136, USA, [3]State Key Laboratory of Experimental Hematology, Institute of Hematology & Blood Diseases Hospital, Center for Stem Cell Medicine, Chinese Academy of Medical Sciences & Peking Union Medical College, Tianjin 300020, China, [4]Showa University Fujigaoka Hospital, 1-30 Fujigaoka Aoba-Ku, Yokohamashi Kanagawa, 227-8501, Japan, [5]Human Oncology and Pathogenesis Program, Memorial Sloan Kettering Cancer Center, New York, NY 10065, USA.

[§]These authors contributed equally to this work

*Correspondence and requests for materials should be addressed to F.-C.Y. (fxy37@med.miami.edu) and Y.Z. (yuanzhou@ihcams.ac.cn)

a

Mutation status of *ASXL1* for all MDS/MPN patients

patients	Gender	Diagnosis	Age at diagnosis	ASXL1 status	alteration
M6	M	CMML	66	c.2269C>T;p.Q757X	nonsense
M73	F	CMML	57	normal	
M97	M	CMML	62	c.2535delC; p.S846VfsX21	frameshift
M144	M	CMML	72	normal	
M260	M	CMML	78	c.2929C>T; p.Q977X	nonsense
M277	M	CMML	87	normal	
M288	M	CMML	81	normal	
M319	M	CMML	42	c.1772dupA; p.Y591X	nonsense
M340	F	CMML	63	normal	
M347	M	CMML	29	normal	
M353	F	CMML	63	normal	
M368	M	CMML	61	c.1900-1922del; p.E635Rfs15X	frameshift
M397	F	JMML	12	c.3190-3191delAG; p.S1064LfsX22	frameshift
M429	F	CMML	57	normal	
M432	M	CMML	42	normal	
M479	M	CMML	75	normal	
M503	M	CMML	64	normal	
M545	F	CMML	49	normal	

b

Mayo prognostic risk category (%)

	Low (%)	Intermediate (%)	High (%)
ASXL1 WT	0 (0/12)	66.7 (8/12)	33.3 (4/12)
ASXL1 mut	0 (0/5)	60 (3/5)	40 (2/5)

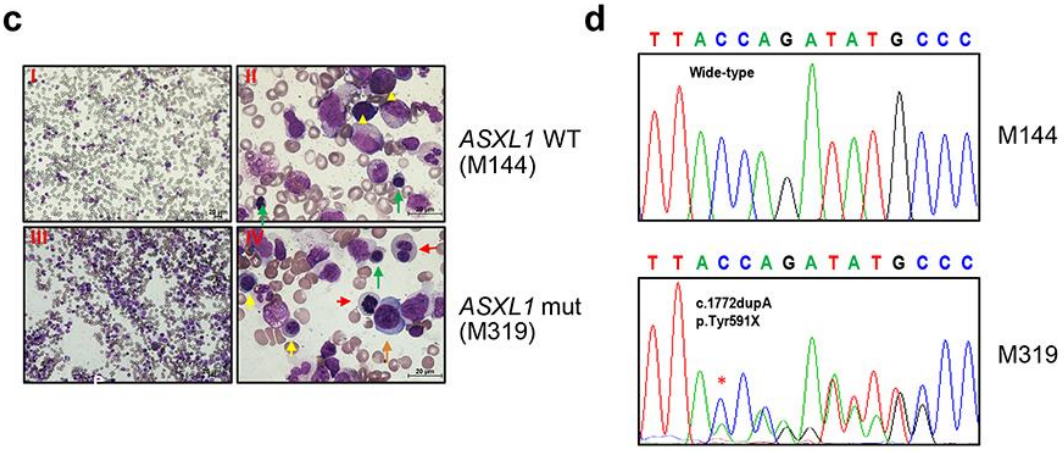

Supplementary Figure S1. More severe anemia in MDS/MPN patients with *ASXL1* mutations. (a) Clinical information and mutation status of *ASXL1* for all MDS/MPN patients (n = 18). **(b)** Mayo prognostic risk category of the 17 patients with CMML. The Mayo Prognostic Scoring System was used for prognostic evaluation and divide patients into three risk groups

(low, intermediate and high) based on the presence of circulating blasts and their absolute monocyte count, hemoglobin and platelet count. **(c)** BM smears from CMML patients with (III, IV) and without (I, II) *ASXL1* mutation were stained with May-Giemsa staining. Representative images of M144 and M319 were taken under 20× (I, III) or 100× (II, IV) magnification. Orange arrows indicate Pro-E, yellow arrows indicate Poly-E, green arrows indicate Ortho-E and red arrows indicate dyserythropoiesis. Scale bar represents 20 μm **(d)** In patient M319, a duplication of an adenine nucleotide (c.1772dupA) was found in the BM cells (mutation site was indicated with red star). Sense sequences are shown.

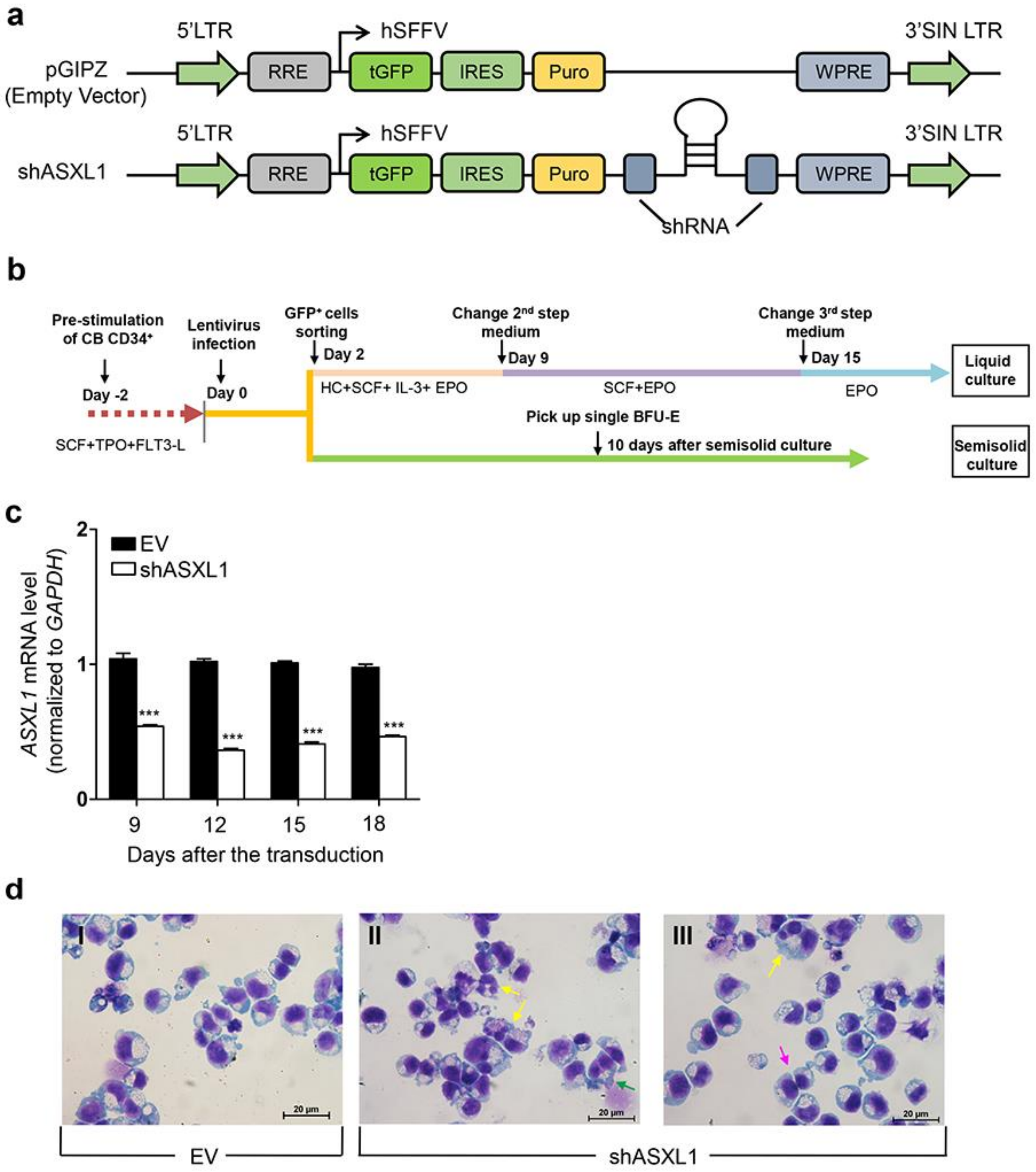

Supplementary Figure S2. Knockdown of *ASXL1* in CB CD34$^+$ cells impairs erythropoiesis and decreases enucleated erythrocytes. (a) Schematic representation of the lentiviral shASXL1 and the empty vector control. **(b)** Timeline of erythroid differentiation in CB CD34$^+$ cells. Before transduction, the CB CD34$^+$ cells were pre-stimulated in StemSpan medium supplemented with human SCF (100 ng/mL), Flt-3L (100 ng/mL) and TPO (50 ng/mL) for 48 hours. The cells were then transduced (day 0) with a lentivirus targeting human *ASXL1* or empty vector in the erythroid differentiation culture medium (IMDM basic medium plus 10^{-6} M hydrocortisone, 100 ng/mL

SCF, 3 IU/mL EPO and 5 ng/mL IL-3) for another 48 hours. On day 2, the GFP^+ cells were sorted and split into two parts. One part was cultured in methylcellulose (H4434) supplemented with 10% IMDM basic culture medium for 14 days to determine the colony forming capacity, and another part was continued to culture under the same liquid culture condition for an additional 7 days until Day 9. On day 9, the cells in the liquid culture were transferred into the second step medium, IMDM supplemented with SCF and EPO, until day 15. In the last stage (days 15-18), the erythroblast cells were cultured only in the presence of EPO. **(c)** The expression level of *ASXL1* in GFP^+ cells at different time point was measured by qPCR, and the mRNA levels were normalized to *GAPDH* (***$p < 0.001$). Data are represented as mean ± SEM from three independent experiments. **(d)** Morphological analysis of erythroblast populations by May-Giemsa staining on day 12 of liquid culture. *ASXL1*-KD increased the occurrence of dyserythropoiesis, such as multinuclearity (yellow arrows), nuclear budding (green arrow) and nuclear bridging (pink arrow) compared to the empty vector. Representative images (100×) are shown, scale bar represents 20 µm.

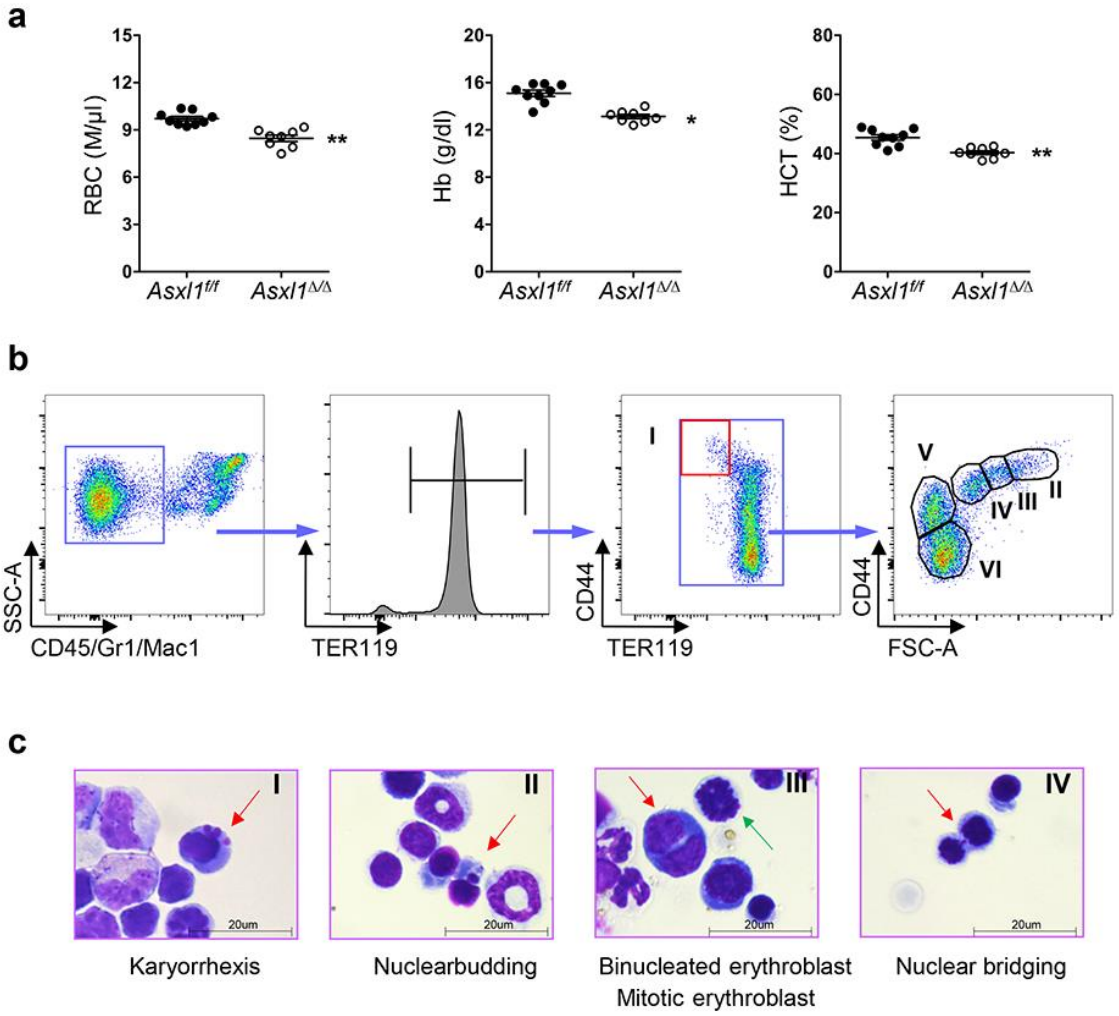

Supplementary Figure S3. Loss of *Asxl1* impairs erythroid terminal maturation in mice. (a) Peripheral blood cell count was performed in *Asxl1*$^{f/f}$ (n = 9 mice) and *Asxl1*$^{\Delta/\Delta}$ (n = 8 mice) at age of 10 months using a Hemavet 500 instrument. Data are presented as mean ± SEM. *$p < 0.05$, **$p < 0.01$. **(b)** Schematic representation of erythroid subsets analysis by flow cytometry. Among live cells, the residual leukocytes, monocytes, and granulocytes were gated out based on CD45, Gr.1 and Mac1. CD45$^-$Gr.1$^-$Mac1$^-$TER119$^+$ cells were analyzed in 2 ways: TER119 versus CD44 and CD44 versus forward scatter (FSC). Population I was gated on the CD44/TER119 plot, and was defined as a CD44^{hi}TER119^{low} population. All other populations were gated on the dot plot of CD44/FSC. **(c)** Representative May-Giemsa stained BM cytospin preparations from *Asxl1*$^{+/-}$ mice are shown. The BM cytospin of *Asxl1*$^{+/-}$ mice showed dysplastic features including karyorrhexis of erythrocytes (I, red arrow), nuclear budding of erythrocytes (II, red arrow), binucleated erythroblast (III, red arrows), mitotic erythroblasts (III, green arrows)

and nuclear bridging in erythrocytes (IV, red arrow). Representative images obtained under 100× magnification are shown, scale bar represents 20 µm.

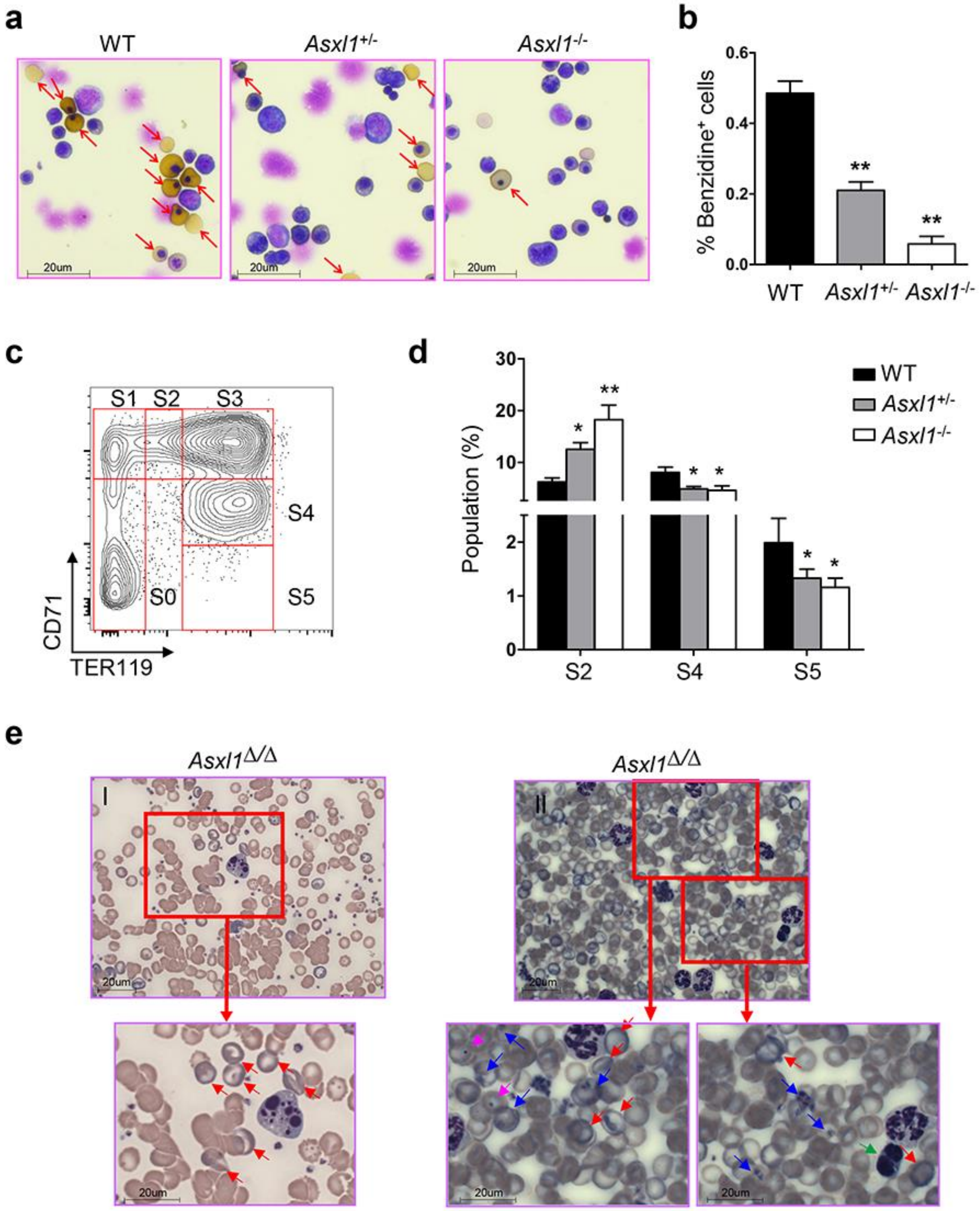

Supplementary Figure S4. Haploinsufficiency of *Asxl1* alters erythroid differentiation *in vivo*. (a) Benzidine- and Giemsa-stained cytospin preparations of cells from the fetal livers of WT, *Asxl1*$^{+/-}$ and *Asxl1*$^{-/-}$ mice. Brown cells are benzidine-positive cells, which are relatively mature cells (red arrow). Representative images were obtained under 40× magnification, scale bar represents 20 µm. **(b)** Quantification of benzidine-positive cells (WT n = 7, *Asxl1*$^{+/-}$ n = 5, *Asxl1*$^{-/-}$ n = 5). Data are presented as mean ± SEM. $**p < 0.01$. **(c)** Schematic representation of

erythroid subset analysis in the fetal liver stained for CD71/TER119 by flow cytometry. **(d)** Quantitation of erythroid populations in the fetal liver of WT, *Asxl1*$^{+/-}$ and *Asxl1*$^{-/-}$ mice using a flow cytometry strategy to determine the distribution of erythroid subsets according to their TER119 and CD71 cell surface expression (n = 6 mice/genotype). Data are presented as mean ± SEM. *$p < 0.05$, **$p < 0.01$. **(e)** May-Giemsa stained PB smears prepared from representative *Asxl1*$^{\Delta/\Delta}$ mice are shown (I, II). PB smears from *Asxl1*$^{\Delta/\Delta}$ mice showed dysplastic features including basophilic erythrocytes (I, II red arrows), nuclear bridging of erythrocytes (II, green arrow), an increased number of polychromatophilic RBCs (II, blue arrows) and Howell-Jolly body in erythrocytes (II , pink arrows). Representative images obtained under 100× magnification are shown, scale bar represents 20 µm.
